# Stability and Anomalous Aggregation in Suspensions of Polymer-Grafted Nanoparticles

**DOI:** 10.1021/acs.macromol.5c02667

**Published:** 2025-11-12

**Authors:** Masoud Abdi, David Amirsadri, Irene Andreu, Ryan Poling-Skutvik

**Affiliations:** † Department of Chemical, Biomolecular, and Materials Engineering, 4260 University of Rhode Island, Kingston, Rhode Island 02881, United States; ‡ Department of Physics, 4260 University of Rhode Island, Kingston, Rhode Island 02881, United States

## Abstract

Polymer-grafted nanoparticles (PGNPs) are a class of hybrid materials with properties intermediate between those of polymer and colloidal systems. Here, we assess how polymer conformation and solvent interactions govern structural stability, phase transitions, and aggregation kinetics in suspensions of gold nanoparticles (AuNPs) grafted with polystyrene of varying molecular weights. When suspended in cyclohexane, the PGNPs exhibit a two-step transition as a function of temperature in which the polymer corona contraction precedes aggregation below the upper critical solution temperature. Time-dependent dynamic light scattering measurements reveal that this aggregation follows a diffusion-limited aggregation process in which the hydrodynamic size increases with time according to a power law with an exponent α ≈ 1/3. This exponent is significantly lower than that observed for colloidal systems. We attribute this discrepancy to a combination of long-range interactions and the viscoelasticity of the grafted layer facilitating rearrangements within the aggregate. Our findings provide quantitative measurements of PGNP phase behavior by quantifying the rate of aggregation, combining polymer thermodynamics and colloidal physics.

## Graphical abstract

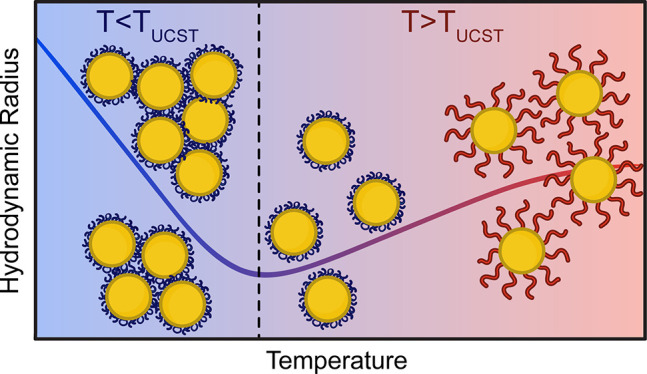

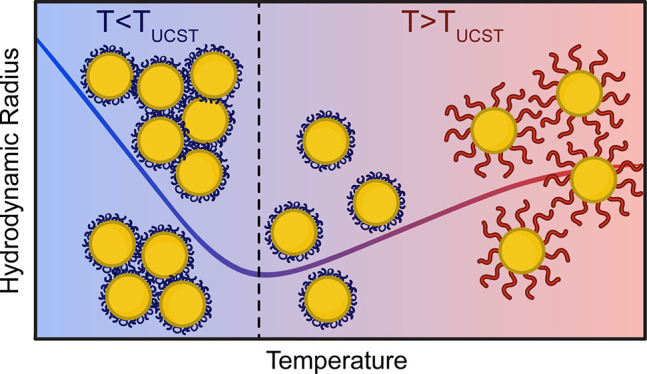

## 1 Introduction

Polymer-grafted nanoparticles (PGNPs) are a class of hybrid material in which linear polymer chains are covalently attached to a nanoparticle core at the chain end, forming a brush architecture.^1–7^ In practice, the polymeric coating enables dispersion and suspension in a wide variety of solvents^3,5^ while the particulate chemistry controls some functionality. Specifically, these materials can be used for plasmonic sensing,^8^ drug delivery,^9,10^ lubrication,^11,12^ catalysis,^13^ and biomedical imaging.^14^ Controlling PGNP functionality requires precision tailoring of dispersion and aggregation. While individual particles may have, for example, well-defined optical and electrical properties,^3,15^ aggregation can cause dramatic shifts in these properties based on interparticle spacing and network formation.^16,17^ Understanding the thermodynamic framework that governs polymer–solvent interaction is therefore crucial for controlling the stability of PGNPs.

A qualitative understanding of PGNP stability in suspension can be developed by considering classical descriptions of polymer solubility and mixing. Polymers in a good solvent exhibit favorable miscibility, where the free energy of mixing Δ*G*_mix_ = Δ*H*_mix_ – *T*Δ*S*_mix_ is negative and dispersion occurs spontaneously. For a linear, Gaussian chain dissolved in a solvent, Flory–Huggins theory^18,19^ describes

$$\Delta G_{\mathrm{mix}}=kT\left[\frac{\varphi }{N}\ln(\varphi )+(1-\varphi )\ln(1-\varphi )+\chi \varphi (1-\varphi )\right]$$ 1

where the first two terms capture the entropy of the mixed system with φ as the volume fraction of polymer and *N* the degree of polymerization and the final term describing the enthalpic interactions between solvent and polymer with an interaction parameter that can be empirically approximated as χ = *A* + *B*/*T*. For an upper critical solution temperature (UCST) system, *B* is positive so that χ increases with decreasing temperature.^20^ According to the Flory–Huggins theory, at high temperatures such as χ < 0.5, the system is in a good solvent condition (i.e., Δ*G*_mix_ < 0). As *T* decreases, however, the solvent quality becomes worse so that χ = 0.5 and Δ*G*_mix_ = 0, which represents the binodal transition temperature. At even lower *T*, the UCST system phase separates as χ > 0.5 and Δ*G*_mix_ > 0. Although this theoretical framing was derived for free chains, it has successfully described the qualitative phase behavior of PGNPs.^21,22^ Quantitative descriptions would require the development of theories that successfully incorporate the conformation and topology of grafted systems.

Beyond Flory–Huggins theory, the chemical identity of these grafted chains helps PGNPs overcome immiscibility challenges^3,23^ and instability in colloidal suspensions. The grafting density, molecular weight, and chain architecture of the grafted chains influence their conformation on the nanoparticle surface and, consequently, the thermodynamics and phase behavior of PGNP solutions.^24,25^ At low grafting densities, polymer chains adopt “mushroom” conformations with minimal interchain interactions, while at intermediate densities, chains begin to overlap and extend from the surface. At high grafting densities, significant chain stretching occurs due to strong excluded volume effects, leading to the formation of highly extended polymer brushes in a dense layer.^3^ The degree of chain stretching and the resulting brush thickness strongly depend on solvent quality. In a good solvent, the polymer canopy is swollen due to favorable polymer–solvent interactions, whereas in poor solvents or near the θ temperature, the canopy collapses, leading to instability and phase separation.^20,26^ For example, solvent quality has been shown to induce microphase separation in solutions^21,27^ or autophobic dewetting when embedded in a linear polymeric matrix.^28–30^ The phase behavior has also been explored for single-polymer component^31–34^ and blends.^35,36^ Although the stability of PGNPs share similarities with that of free polymers,^22^ there are significant differences. Past work has shown that PGNP systems display a suppressed cloud point relative to free chains,^21^ consistent with observed trends for polymers with complex architectures.^37–39^

Here, we resolve differences between PGNP phase behavior in a UCST system and that of analogous polymeric and colloidal systems by precisely defining and characterizing the phase boundary and aggregation rates. Using dynamic light scattering, we find that decreasing solvent quality induces corona collapse, driving PGNP aggregation with distinct and anomalous kinetics. Our findings demonstrate that PGNP phase behavior can be better understood through polymer thermodynamics rather than colloidal physics. Through a new kinetic dynamic light scattering acquisition protocol, we observe that the PGNP aggregates grow in size as a power-law in time with an exponent that exhibits a step change from 0 to approximately 1/3 at the binodal temperature. This aggregation rate contrasts with the universal power-law growth observed for hard-sphere colloids with short-range attractions. Furthermore, by defining phase separation according to the *rate* of aggregation, we remove thermal history effects and other ambiguities, enabling a precise determination of *T*_UCST_. Our findings thereby elucidate how grafted conformations modify polymer thermodynamics and facilitate improved predictions of the phase behavior of grafted systems.

## 2 Materials and Methods

Sodium citrate tribasic dihydrate (Na_3_C_6_H_5_O_7_·2H_2_O, 99%), gold­(III) chloride trihydrate (HAuCl_4_·3H_2_O, 99.9%), and *N*,*N*-Dimethylformamide (DMF, 99%) were purchased from Sigma-Aldrich. Tetrahydrofuran (THF, 99%) and cyclohexane (99%) were obtained from VWR, Inc. Thiol-terminated polystyrene (PS-SH) with weight-average molecular weight *M*_w_ of 5.8 kDa (dispersity Đ = *M*_w_/*M*_n_ = 1.1), 27 kDa (Đ = 1.07), 61 kDa (Đ = 1.08), and 259 kDa (Đ = 1.11) was purchased from Polymer Source and denoted as PS5.8, PS27, PS61, and PS259, respectively. All experiments were conducted using ultrapure water (24 MΩ cm) obtained from a Millipore Milli-Q system.

### 2.1 Synthesis of Gold Nanoparticles (AuNPs)

AuNPs with an average radius of *R* = 7.3 ± 2.1 nm (*n* = 1200 particles) were synthesized using a seed-growth method using a modified Turkevich protocol.^40–42^ Briefly, 1.05 mL of gold­(III) chloride solution (50 mM) was diluted with 91.95 mL water in a round-bottom flask. The solution was heated to 95 °C under continuous stirring, and then 7 mL of sodium citrate solution (10 mg mL^–1^) was rapidly added into the flask. The reaction mixture was maintained at 95 °C with constant stirring for 40 min, during which the color changed from light yellow to deep red indicating successful AuNP formation. The solution was then cooled to room temperature and stored at 4 °C for further use. The concentration of AuNPs was determined to be 0.082 ± 0.01 mg mL^–1^ from the Beer–Lambert law using UV–vis extinction spectroscopy with an extinction coefficient ϵ = 3.9 × 10^8^ M^–1^ cm^–1^.^43^

### 2.2 Functionalization of AuNPs with Thiol-Terminated Polystyrene (PS-SH)

AuNPs were first centrifuged and redispersed in DMF and then added dropwise into a DMF solution of PS5.8, PS27, PS61, and PS259 kDa at a polymer concentration of 0.7 mg mL^–1^ under rapid stirring. DMF was chosen as the initial solvent because its high polarity ensures that citrate-coated AuNPs remain stable after transfer from aqueous media, while also providing good solvation of the polymer chains. After stirring overnight at room temperature, a solution of THF and polymer (0.7 mg mL^–1^) was added to the mixture and allowed to react for another 24 h. THF was introduced in the second step because it is a better solvent for polystyrene and promotes efficient grafting onto the nanoparticle surface. The final product was purified through three successive cycles of centrifugation–redispersion at 8000, 14,000, and 20,000 RCF, respectively, to remove any unreacted polymer. The purified PGNPs were suspended in THF for TGA, UV–vis, and TEM measurements. For DLS measurements, the solutions were centrifuged and redispersed in cyclohexane.

### 2.3 Thermal Gravimetric Analysis (TGA)

TGA samples were prepared by pipetting PGNP solutions onto a clean platinum pan. The samples were then dried in a vacuum oven to evaporate the solvent. Measurements were conducted on a TA Instruments TGA 55 over a temperature range 25–600 °C at a heating rate of 5 °C min^–1^. The polymer grafting density σ was calculated according to

$$\sigma_{\mathrm{TGA}}=\frac{m_{\mathrm{polymer}}\rho_{\mathrm{core}}R_{\mathrm{core}}N_{A}}{m_{\mathrm{core}}3M_{W}}$$ 2

where *m*_polymer_ and *m*_core_ are the polymer and core masses, respectively, ρ_core_ is the core density, *R*_core_ is the core radius, and *N*_A_ is Avogadro’s number, as described in literature.^15,44^ The *m*_polymer_ was determined at 200 °C to eliminate any contributions from residual THF. The AuNP core radius *R*_core_ was determined by TEM as described below. The polymer graft density σ varied among the samples, with values of 1.29 ± 0.35, 0.53 ± 0.14, 0.64 ± 0.18, and 0.07 ± 0.02 chains nm^–2^ for PS5.8, PS27, PS61, and PS259, respectively.

### 2.4 UV–Visible (UV–Vis) Spectroscopy

PGNP solutions at an appropriate concentration (0.03–0.05 mg mL^–1^) were loaded into a quartz cuvette with a path length of 1 cm. UV–vis extinction spectra were recorded using a Shimadzu UV-2600i spectrophotometer at a scanning rate of 225 nm min^–1^ over a range of 400–900 nm.

### 2.5 Dynamic Light Scattering (DLS)

DLS measurements were conducted on PGNP samples at a concentration of 0.01–0.02 mg mL^–1^ using a Brookhaven BI-200SM instrument with a wavelength of λ = 640 nm, and 40 mW diode laser as the light source. A scattering angle of 90° was used to measure the scattered light intensity. Additionally, measurements at high temperature (i.e., 50 °C) were conducted at four different scattering angles, ranging from 40° to 120°. The intensity autocorrelation function *G*_2_ was fit to a stretched exponential function given by

$$G_{2}(q,\tau )=A+B\exp[-2(\Gamma \tau {)}^{\beta }]$$ 3

where τ is the delay time, Γ is the relaxation rate, and β is a stretching exponent that characterizes the particle size dispersity. *A* and *B* are constants that characterize the signal noise at long times and the signal amplitude at short times, respectively. The PGNP diffusivity is then determined by *D* = Γ/*q* ^2^, where *q* = 4π*n*sin­(θ/2)/λ is the wavevector and *n* = 1.426 is the index of refraction of cyclohexane. The PGNP hydrodynamic radius is finally calculated by the Stokes–Einstein equation *R*_H_ = *k*_B_ *T*/6πη*D* where *k*_B_ is the Boltzmann constant, η is the temperature-dependent solvent viscosity, and *T* is the temperature.

### 2.6 Transmission Electron Microscopy (TEM)

The PGNP nanostructures were analyzed using transmission electron microscopy (TEM, JEOL JEM-F200) operated at an accelerating voltage of 200 kV. TEM samples were prepared by drop-casting 3 μL of suspension onto Formvar/carbon-coated 400-mesh copper grids (Electron Microscopy Sciences), which were left to dry at room temperature and ambient pressure. From the TEM images, we identified particles using a threshold-based contrast algorithm and manually measured the smallest surface-to-surface distance particles at each surface functionalization condition to determine the height of the grafted polymer layers.

## 3 Results and Discussion

We conducted an initial characterization of the polystyrene-functionalized AuNPs using TEM, as shown in Figure 1. In good agreement with previous literature reports,^45^ the AuNP cores are have a radius of *R*_NP_ = 7.3 ± 2.1 nm. After functionalization with PS-SH, the AuNP cores are separated by the grafted polymer coronas, and the distance between cores increases with increasing *M*_w_, indicating that the corona thickness *h* increases with *M*_w_. The TEM imaging demonstrates that most AuNPs have been successfully functionalized with polystyrene of different *M*_w_ with the described methods.

**Figure 1:**
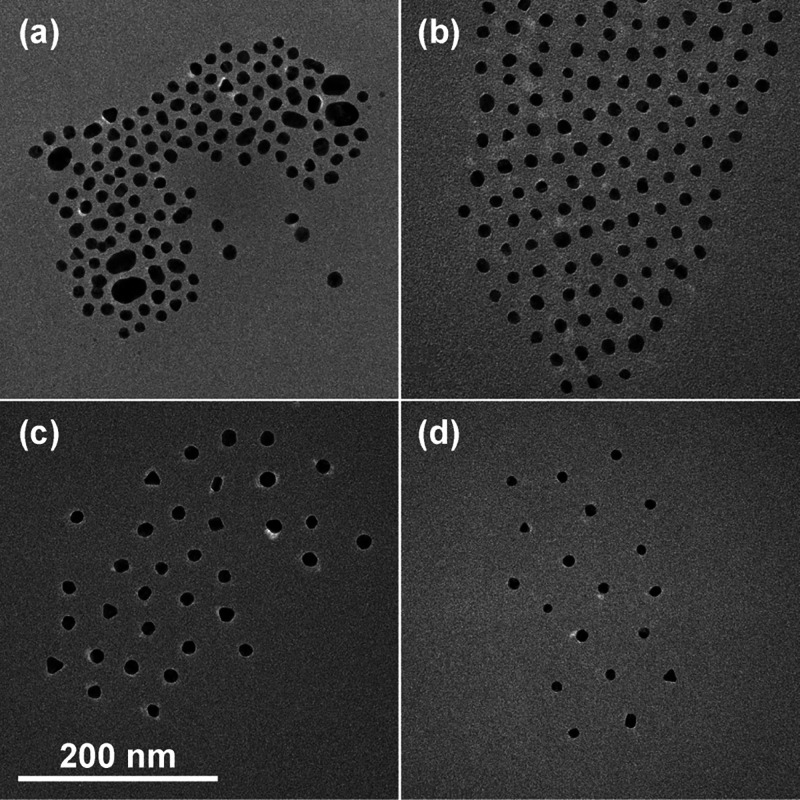
TEM images of AuNPs functionalized with polystyrene with molecular weights of (a) 5.8 kDa, (b) 27 kDa, (c) 61 kDa, and (d) 259 kDa.

To confirm that the polystyrene coating stabilizes the AuNPs in organic solvents, we measure the extinction spectra of dilute solutions of PGNPs at a concentration of ≈0.04 mg mL^–1^ dissolved in THF, as shown in Figure 2. Bare AuNPs were measured as an aqueous suspension. As expected for nanoscale gold, we observe that the bare AuNPs exhibit a single peak at a wavelength of λ = 520 nm. This peak corresponds to the localized surface plasmon resonance (LSPR) signal of the gold nanoparticle core and is consistent with the particle size measured in TEM.^46^ For PGNPs, we observe a subtle red shift of the LSPR peak to λ = 526 nm as a result of changes to the electrical permittivity of the solvent and changes to the local refractive index near the nanoparticle surface from the grafted polymer chains.^47–49^ The consistency of this primary peak across different polymer *M*_w_ indicates that the vast majority of AuNPs exist as individual PGNPs, in agreement with the observations from TEM. These characterization results verify the successful functionalization of AuNPs with polystyrene chains of varying *M*_w_ and their dispersion within a good solvent.

**Figure 2:**
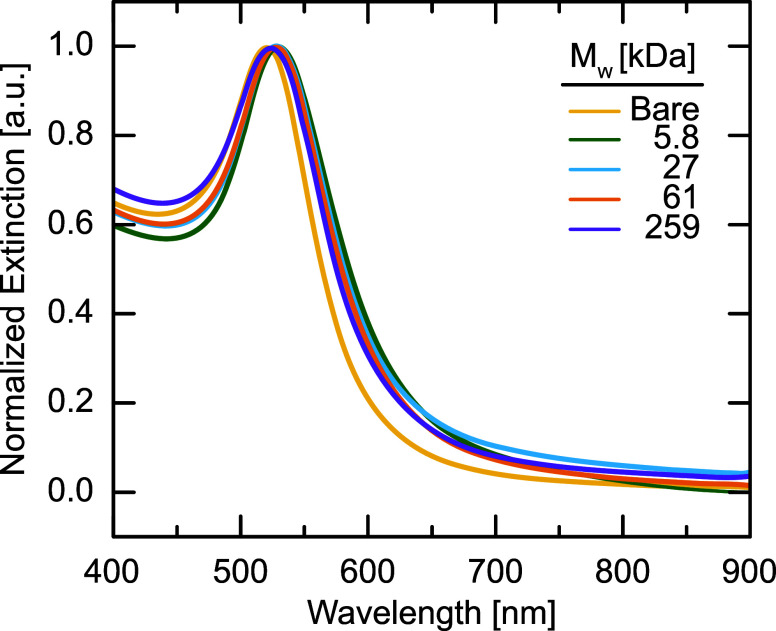
UV–vis extinction spectra of bare and PGNPs normalized to the height of primary peak.

Although extinction spectroscopy confirms that the AuNPs remain dispersed within organic solvents, it does not provide information on the conformation of the grafted chains. We, therefore, use DLS to measure the dynamics of the PGNPs in dilute suspension and infer the thickness of the grafted corona. We measure the intensity autocorrelation function *G*_2_ of PGNPs with polymers of varying *M*_w_ as a function of delay time τ (Figure 3a). Bare particles exhibit a fast relaxation due to their small size, but these relaxations dramatically slow for PGNPs, indicating an increase in their effective size. To quantify this shift, we fit *G*_2_ to a stretched exponential (eq 3) to extract the relaxation rate Γ, and convert this rate into an effective hydrodynamic radius *R*_H_ using the Stokes–Einstein expression. We find the *R*_H_ of PGNPs functionalized with PS5.8, PS27, PS61, and PS259 to be 13.3 ± 0.3, 18.4 ± 0.8, 31.7 ± 1.5, and 66.0 ± 4.0 nm, respectively in cyclohexane at 50 °C. By subtracting the size of bare AuNPs, we determine the thickness *h* of the grafted polymer corona, which we compare as a function of *M*_w_ to values determined from TEM (Figure 3b). First, we find that *h* of the grafted corona is consistently larger when measured in DLS than in TEM because the polymer swells in a good solvent. Second, we find that *h* follows a power-law scaling with *M*_w_ according to *h* ∼ *M*_w_^δ^ with δ = 0.63 ± 0.07 and 0.46 ± 0.05 for DLS and TEM measurements, respectively. These scaling exponents indicate that the grafted polymer adopts more extended conformations when dispersed in the solvent, as measured with DLS, than in the melt state, as measured with TEM. Theoretical derivations^50,51^ predict that *h* scales with *M*_w_ with δ = 1 in planar polymer brush systems. Spherical geometries, however, introduce a radial dependence to monomer concentration, leading to a transition from a concentrated polymer brush (CPB) regime near the particle surface to a semidilute polymer brush (SDPB) regime at longer distances.^52–54^ This structural change in the polymer brush results in a transition from δ = 1 in CPB to δ = 0.6 in SDPB.^55^ The grafted height should also depend on grafting density according to *h* ∼ σ^1/2^ in CPB and *h* ∼ σ^1/3^ in SDPB.^54^ We note that our grafting density decreases with increasing *M*_w_ because the larger chains generate larger steric repulsions and therefore our measured power-law scaling of *h* reflects contributions from both σ and *M*_w_. Our scaling exponent δ = 0.63 ± 0.07 is therefore consistent with existing literature and suggests that a majority of grafted chains exist within the SDPB regime. Although full characterization of this conformation would require more precise measurements, such as small angle neutron scattering,^52^ our results confirm that these particles are effectively grafted with polymer and stable within organic solutions.

**Figure 3:**
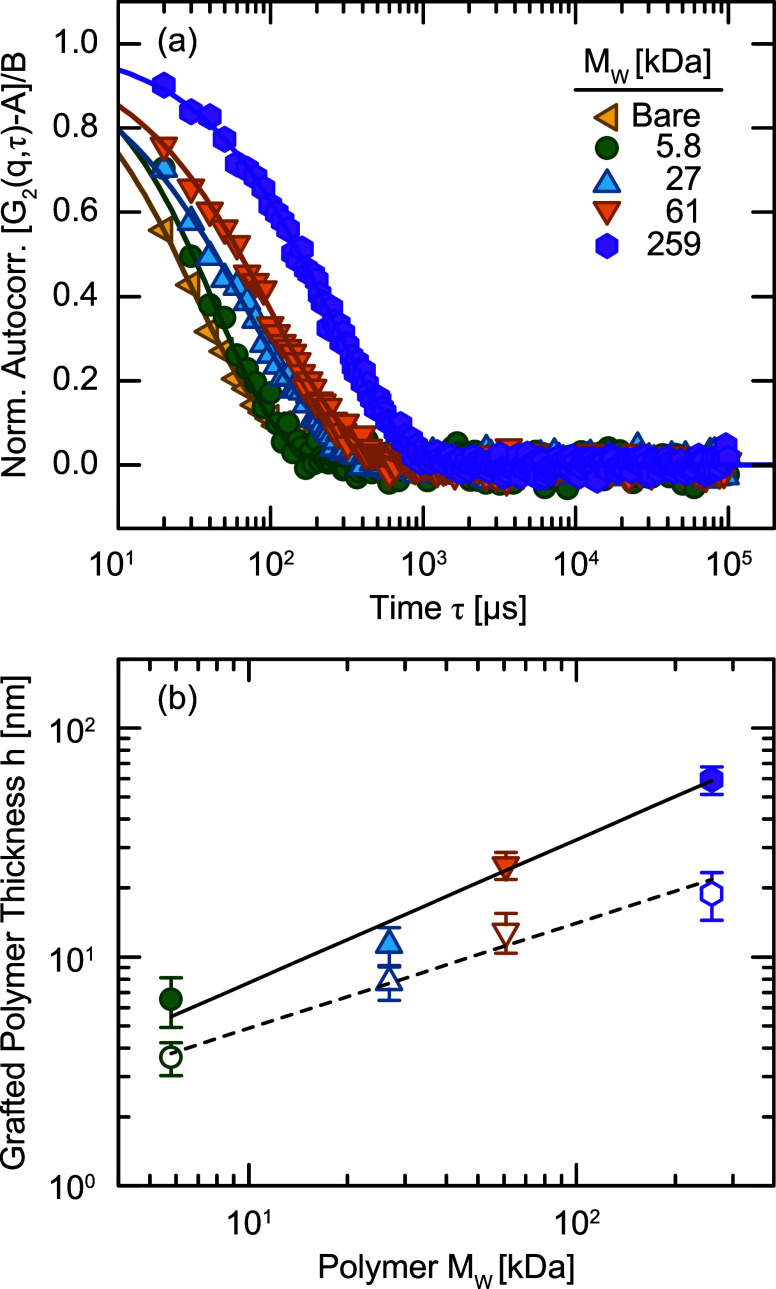
(a) Normalized intensity autocorrelation functions *G*_2_(*q*, τ) for PGNPs of varying *M*_w_ at *q* = 19.8 μm^–1^. (b) Grafted polymer thickness *h* determined by DLS (closed symbols) and TEM (open symbols) as a function of grafted molecular weight. Solid and dashed lines represent power-law fits *h* ∼ *M*_w_^δ^ with δ = 0.63 ± 0.07 and 0.46 ± 0.05 for DLS and TEM data, respectively.

We now characterize how the grafted polymer conformation varies with solvent quality by dispersing the PGNPs in cyclohexane, which forms a UCST system with polystyrene.^56^ To characterize the phase behavior of PGNPs, we capture DLS measurements at different temperatures from 50 to 7 °C, just above the freezing point of cyclohexane, at a rate of approximately 0.1 °C min^–1^ and measure the resulting change in *R*_H_ (Figure 4). We normalize *R*_H_ by the value at 50 °C to easily compare the behavior across grafted *M*_w_. At high *T*, the PGNPs are stable in solution with a swollen corona as expected from the good solvent condition. As the temperature decreases, the solvent quality worsens and we observe a gradual decrease in the effective size as the grafted chains contract toward the particle surface. This contraction occurs because the grafted chains are stretched away from the particle surface in solution as confirmed by the scaling of *R*_H_ with *M*_w_ (Figure 3b), requiring a gain in enthalpic interactions to offset the reduction in conformational entropy. As the solvent quality worsens, the grafted chains no longer experience sufficient enthalpic interactions with the solvent and must regain some entropy. As a result, the corona collapses into a more compact conformation, causing a decrease in *R*_H_ as shown in Figure 4. We observe that PGNPs with PS27, PS61, and PS259 experience a maximum contraction to ∼80% of their swollen size. With this relatively limited data set, we cannot comment on whether this observation is a universal property of grafted particle systems or simply a coincidence for these experimental parameters. We do not see any significant change in the size of PGNPs grafted with PS5.8, which is likely a result of their small size making changes insignificant relative to experimental error. For example, an 80% contraction in size would result in less than a 3 nm change to the radius, which is well within our experimental error.

**Figure 4:**
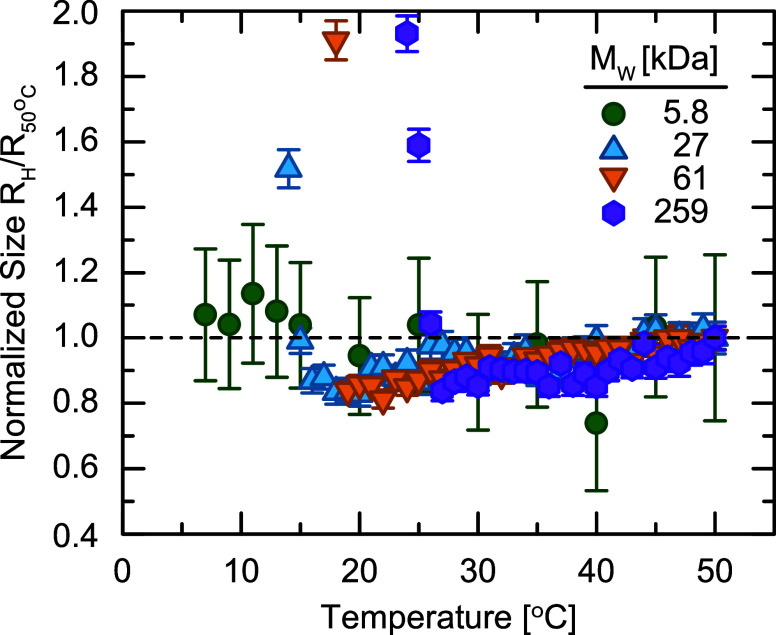
Hydrodynamic radius *R*_H_ of PGNPs with various *M*_w_ measured as a function of temperature at a cooling rate of 0.1 °C min^–1^ starting at 50 °C. Size is normalized to *R*_H_ at 50 °C for clarity.

At sufficiently low temperatures, we observe that the normalized size of the PGNPs measured in DLS experiences a dramatic increase. Such an increase in measured size indicates that the PGNPs are destabilized and beginning to aggregate. This aggregation must be initiated by unfavorable interactions between the polymer and solvent, which can be minimized beyond the full collapse of the corona through interparticle association. Thus, in analogy to phase separation in polymer solutions, the onset of aggregation determines the binodal temperature for the PGNP suspension, allowing us to define the phase transition temperature *T*_UCST_ as the first temperature at which the size becomes larger than *R*_H_ at 50 °C (Table 1). This onset temperature increases with increasing *M*_w_, consistent with theoretical descriptions for free polymer.^20^ We note, however, that aggregate size depends strongly on the measurement time scale. First, under a temperature ramp, there are temperature gradients in the DLS chamber and within the sample. Second, aggregation is a kinetic process, making *R*_H_ inherently dependent on measurement time. Together, these effects make the increase in effective *R*_H_ an imprecise measurement of the binodal transition.

**Table 1:** Comparison of Three Different Methods for Defining *T*_UCST_ of PGNPs with Various *M*_w_ ^a^

	*T*_UCST_ (°C)			
*M*_w_ (kDa)	**linear PS** ^56,70^	ramp	quench	aggregation α
27	9–10	13	15	16
61	16–18	18	19	19
259	25–26	25	27	27

a Values for linear polystyrene in cyclohexane are estimated from the cited literature.

To demonstrate this ambiguity in aggregate size at temperatures below the binodal, we conduct an alternative experiment in which we directly quench the samples from 50 °C to varying temperatures and measure the hydrodynamic size of PGNP aggregates after 2 h (Figure 5). While the temperature ramp results in a continuous, gradual increase in aggregate size with temperature, the aggregate size following quenches exhibits a rapid and discontinuous step change for all *M*_w_. These step changes define the phase transition at slightly higher temperatures than what was observed during ramps, confirming that aggregate size is a kinetically controlled property. At low temperatures, these different measurements of PGNP aggregate size converge to similar values, which likely reflects nearly complete aggregate formation and therefore depends on the total concentration of PGNPs in solution rather than temperature.

**Figure 5:**
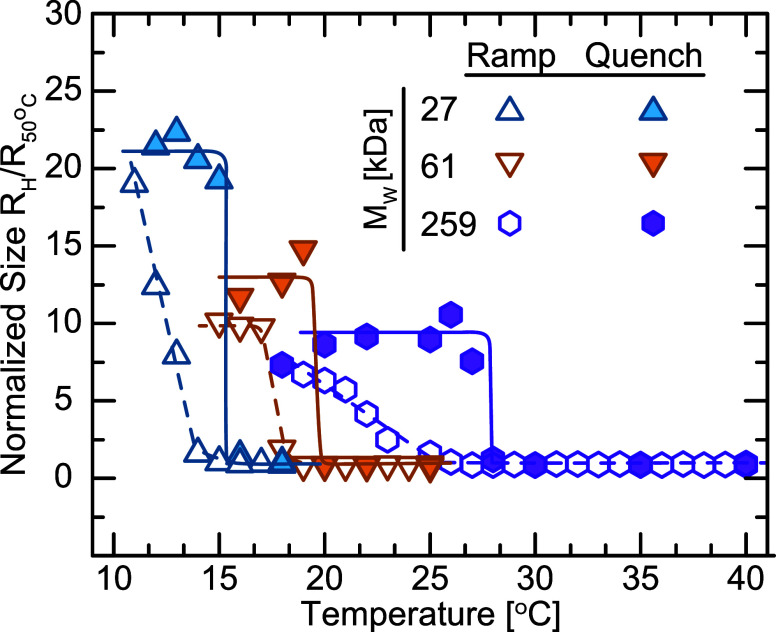
Hydrodynamic radius *R*_H_ of PGNPs measured with various molecular weights *M*_w_ as a function of temperature, normalized to *R*_H_ at 50 °C. The data are shown for temperature ramps at a rate of 0.1 °C min^–1^ (open symbols) and quenches (closed symbols). The lines serve as guides to the eye.

Because aggregate size is not solely a function of *T* but also depends strongly on the thermal history, we propose an alternative experimental approach to quantify the rate of aggregation following thermal quenches from 50 °C to determine a time-independent metric of phase separation. Similar approaches were used in classical polymer blend studies, where small-angle neutron scattering was analyzed as a function of time after thermal quenches to extract thermodynamic interaction parameters.^57,58^ We measure the hydrodynamic size of PGNPs aggregates as a function of time at various quench temperatures, as shown in Figure 6. At *T* > *T*_UCST_ (e.g., 28 °C for PS259), the effective size remains constant over time, indicating that the PGNPs are stable in solution. For lower *T*, however, the aggregate size grows over time as a power-law according to *R*_H_ ∼ *t* ^α^. This power-law growth exists for all quench depths with *T* < *T*_UCST_, suggesting that once the grafted corona has collapsed, these aggregates grow through a diffusion-limited aggregation (DLA) process.^59–62^ Furthermore, we observe qualitatively similar power-law growth for PGNPs with different *M*_w_ at a constant quench depth Δ*T* = *T* – *T*_UCST_ = – 5 °C. The ubiquity of this power-law growth indicates that PGNPs undergo aggregation similar to colloidal systems and independent of grafted *M*_w_.

**Figure 6:**
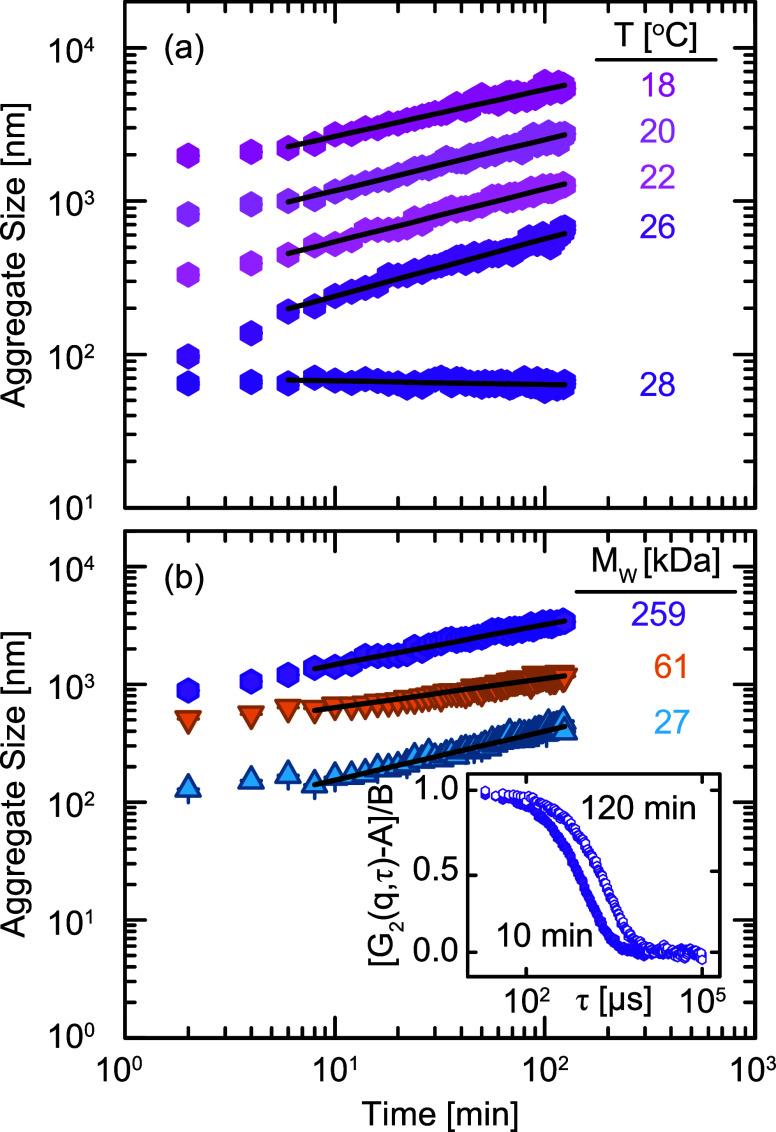
(a) Hydrodynamic radius of 259 kDa-PGNP aggregates as a function of time at different quench temperatures. Curves at 26 °C, 22 °C, 20 °C, and 18 °C are shifted by 1.2, 2.6, 6, and 14 for temperatures decreasing from 26 to 18 °C, respectively, for visual clarity. (b) Hydrodynamic radius of PGNP aggregates of varying *M*_W_ at a constant quench depth Δ*T* = – 5 °C. Curves of 61 kDa, and 259 kDa shifted vertically by 5 and 7, respectively, for visual clarity. Solid lines represent power-law fits to the growth trends. Inset: Normalized intensity autocorrelation function *G*_2_(*q*, τ) for 259 kDa at *q* = 19.8 μm^–1^ after 10 and 120 min quenched at 22 °C.

To quantify the rate of aggregation, we extract the power-law exponent α and plot as a function of *T* (Figure 7). We notice two important characteristics. First, α exhibits a rapid change from α = 0 to α ≈ 1/3 at a temperature that depends on *M*_w_. This step change is consistent with a first-order transition, which is typically characterized by a discontinuity in a fundamental property,^63^ between the 1- and 2-phase regions. Thus, we can precisely define *T*_UCST_ as the highest temperature at which α > 0. Because this exponent is constant with time, our definition of the binodal is now time-independent. We report these values for *T*_UCST_ in Table 1, and find that they agree well with our the observed changes in *R*_*H*_. Additionally, all of our measurements are consistently higher than literature values for free chains with comparable molecular weights, consistent with our earlier studies on PGNP phase separation^21^ and literature reports for polymers with complex architectures.^64–69^

**Figure 7:**
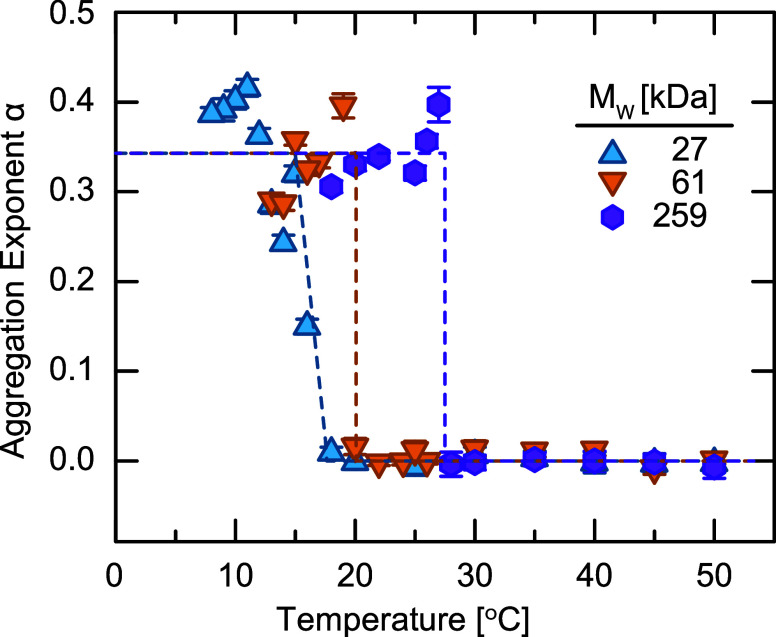
Aggregation exponent α as a function of temperature for PGNPs with different molecular weights. Dashed lines are guides to the eye, indicating the onset of aggregation.

The similar phase boundaries of PGNPs and free chain analogues indicates that PGNP stability is largely controlled by the polymer thermodynamics. In contrast to free chains, however, PGNPs possess a composite structure in which the grafted chains are attached to a hard colloidal core, which may play a significant role in the aggregation process. To understand potential colloidal contributions to PGNP aggregation, we note that for *T* < *T*_UCST_, all of the PGNPs exhibit α ≈ 1/3 within experimental error. The observed power-law growth is therefore independent of grafted polymer *M*_w_ and suggests that the aggregation mode may be primarily controlled by the colloidal nature of these particles. Existing literature on the aggregation of hard-sphere colloids with short-range attractions find similar power-law scalings in the DLA regime,^62^ but with a dramatically different exponent of α ≈ 0.55. In DLA, the aggregation exponent α ≈ 1/*d*_f_, where *d*_f_ is the fractal dimension of the aggregate. Therefore, the smaller value of α observed for PGNPs suggests that the resulting aggregates are significantly denser than those formed in colloidal systems.

We confirm that these PGNP aggregates are dense and approximately spherical through TEM images taken on samples prepared below *T*_UCST_, as shown in Figure 8. In these micrographs, we observe aggregate structures with a compact structure and a smooth interface, in strong contrast to the fractal, diffuse morphologies observed for aggregates of hard sphere colloids or nanoparticles. We hypothesize that this difference between the aggregation rates of hard-sphere colloids and PGNPs is attributable to two potential factors. First, the polymer-mediated interactions are long-range, quantified by the grafted polymer thickness relative to radius of nanoparticle core 0.75 < *h*/*R*_NP_ < 15, as compared to the short-range electrostatic and van der Waals interactions for colloidal systems with κ^–1^/*R*_NP_ < 0.02, where κ^–1^ represents the Debye screening length of interactions.^62,71,72^ The long-range interactions of the grafted polymer chains may improve the efficiency by which PGNPs explore the free energy landscape, facilitating bonding to more attractive sites and increasing the density of the resulting aggregates. Second, the viscoelasticity of grafted polymers may allow particles to rearrange within the aggregate. Whereas hard-sphere colloids are immobilized by the strong bonds between particles in the aggregate, the collapsed grafted layer forms a polymer melt at the particle surface that may relax into the globular conformations with *d*_f_ ≈ 3 observed in phase-separated polymer solutions.^73–76^ This collapsed state minimizes the contact area between polymer chains and free solvent, allowing the system to reach even lower energy states than exist for diffuse fractal-like structures. Although our results do not provide direct mechanistic information on the aggregation process of PGNPs, we have identified essential characteristics of PGNP phase separation that are different than simple polymers or hard-sphere colloidal systems: the onset of aggregation is controlled by polymer–solvent interactions, the aggregation occurs through a DLA process, and the rate of aggregation is suppressed relative to that of hard-sphere colloids due to forming denser aggregates.

**Figure 8:**
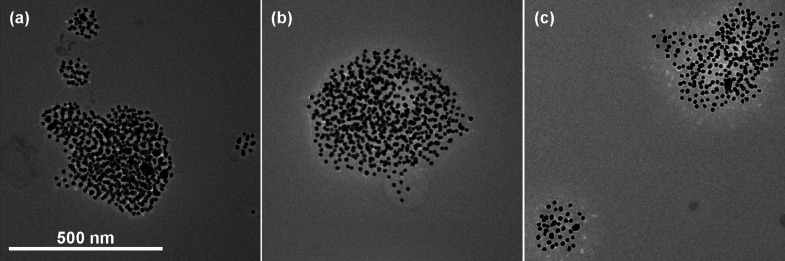
TEM images of aggregated AuNPs functionalized with polystyrene formed below *T*_UCST_ with molecular weights of (a) 27 kDa, (b) 61 kDa, (c) 259 kDa.

## 4 Conclusions

In this paper, we demonstrate the aggregation and dispersion behavior of PGNPs in thermal solvents by varying the molecular weight of the grafted polymers. UV–vis extinction spectroscopy, DLS, and TEM experiments were performed to confirm the presence of a polymer canopy that stabilizes the PGNPs within organic solvents. By conducting DLS experiments at different temperatures, we observed the emergence of a two-phase region below the UCST and precisely identified the binodal temperature according to the power-law aggregation exponent α, which exhibits a discontinuous step change at *T*_UCST_. This definition removes the ambiguity over determining phase behavior through kinetically dependent parameters such as aggregate size, and results in phase transition temperatures that are comparable to but higher than those reported for free chains with similar *M*_w_. Future work will focus on remaining open questions about the mechanism of PGNP aggregation, to test our hypothesis on the contributions of viscoelastic rearrangements and long-range interactions in forming dense, spherical aggregates, and the role of polymer grafting density, particle size, and PGNP concentration in shifting the phase boundary. The unique aggregation behavior of PGNPs may present an opportunity to control and tune the photothermal and optical responses of AuNP aggregates, and we therefore expect our findings to be useful in controlling nanoscale self-assembly.

## References

[1] Francis R., Joy N., Aparna E. P., Vijayan R. (2014). Polymer Grafted Inorganic Nanoparticles,
Preparation, Properties, and Applications: A Review. Polym. Rev..

[2] Kumar S. K., Jouault N., Benicewicz B., Neely T. (2013). Nanocomposites with
Polymer Grafted Nanoparticles. Macromolecules.

[3] Hore M. J. A., Korley L. T. J., Kumar S. K. (2020). Polymer-Grafted
Nanoparticles. J. Appl. Phys..

[4] Hore M. J. A. (2019). Polymers
on Nanoparticles: Structure & Dynamics. Soft Matter.

[5] Chancellor A. J., Seymour B. T., Zhao B. (2019). Characterizing Polymer-Grafted Nanoparticles:
From Basic Defining Parameters to Behavior in Solvents and Self-Assembled
Structures. Anal. Chem..

[6] Sevening J. N., Nupnar N., Adhikary S., Reifsnyder Hickey D., Swulius M. T., Koerner H., Hore M. J. A., Hickey R. J. (2024). From Fully
Stretched to Collapsed Chains: Bottlebrush Polymer Grafted Particles. Macromolecules.

[7] Kumar S. K., Benicewicz B. C., Vaia R. A., Winey K. I. (2017). *50th Anniversary
Perspective*: Are Polymer Nanocomposites Practical for Applications?. Macromolecules.

[8] Vasileiadis T., Dhiman A. K., Noual A., Sbalbi N., Ye M., Macfarlane R. J., Graczykowski B., Fytas G. (2025). Acoustoplasmonic Metasurfaces
Based on Polymer-Grafted Nanoparticles. Nano
Lett..

[9] Pelaz B. (2017). Diverse Applications of Nanomedicine. ACS Nano.

[10] Lahmadi S., Alamery S., Beagan A., Alotaibi K., Alswieleh A. (2024). Advanced Hybrid
Silica Nanoparticles with pH-responsive Diblock Copolymer Brushes:
Optimized Design for Controlled Doxorubicin Loading and Release in
Cancer Therapy. RSC Adv..

[11] Liu J., Qian Y., Li D., Wu W., Zhang M., Yan J., Li B., Zhou F. (2024). Oil-Soluble
Polymer Brushes-Functionalized
nanoMOFs for Highly Efficient Friction and Wear Reduction. Friction.

[12] Peng J., Tang W., Jiang Z., Wang B., Xiao D., He Y., Mou Z. (2024). Synthesis of Oil-Soluble
Polymer-Grafted Carbon Dots
with Outstanding Colloidal Stability and Tribological Properties in
Polyalphaolefin. Langmuir.

[13] Ye S., Wang F., Zhang L., Fan F., Zhang X., Wang T., Fu Y. (2025). Polymer Brush-Grafted
Metal–Organic
Framework Nanoplates for Enhanced Catalysis of CO_2_ Cycloaddition
with Epoxides. Inorg. Chem..

[14] Hou Z., Liu Y., Xu J., Zhu J. (2020). Surface Engineering of Magnetic Iron
Oxide Nanoparticles by Polymer Grafting: Synthesis Progress and Biomedical
Applications. Nanoscale.

[15] Tawade B. V., Singh M., Apata I. E., Veerasamy J., Pradhan N., Karim A., Douglas J. F., Raghavan D. (2023). Polymer-Grafted
Nanoparticles with Variable Grafting Densities for High Energy Density
Polymeric Nanocomposite Dielectric Capacitors. JACS Au.

[16] Haley J. D., Iacovella C. R., Cummings P. T., McCabe C. (2015). Examining the Aggregation
Behavior of Polymer Grafted Nanoparticles Using Molecular Simulation
and Theory. J. Chem. Phys..

[17] Hore M. J., Composto R. J. (2013). Strategies for Dispersing,
Assembling, and Orienting
Nanorods in Polymers. Current Opinion in Chemical
Engineering.

[18] Huggins M. L. (1941). Solutions
of Long Chain Compounds. J. Chem. Phys..

[19] Flory P. J. (1942). Thermodynamics
of High Polymer Solutions. J. Chem. Phys..

[20] Rubinstein, M. ; Colby, R. H. Polymer Physics; Oxford University Press: New York, 2003.

[21] Mongcopa K. I. S., Poling-Skutvik R., Ashkar R., Butler P., Krishnamoorti R. (2018). Conformational
Change and Suppression of the Θ-Temperature
for Solutions of Polymer-Grafted Nanoparticles. Soft Matter.

[22] Izor S., Schantz A., Jawaid A., Grabowski C., Dagher T., Koerner H., Park K., Vaia R. (2021). Coexistence
and Phase Behavior of Solvent–Polystyrene-Grafted Gold Nanoparticle
Systems. Macromolecules.

[23] Choudhury S., Agrawal A., Kim S. A., Archer L. A. (2015). Self-Suspended Suspensions
of Covalently Grafted Hairy Nanoparticles. Langmuir.

[24] Modica K. J., Martin T. B., Jayaraman A. (2017). Effect of
Polymer Architecture on
the Structure and Interactions of Polymer Grafted Particles: Theory
and Simulations. Macromolecules.

[25] Midya J., Rubinstein M., Kumar S. K., Nikoubashman A. (2020). Structure
of Polymer-Grafted Nanoparticle Melts. ACS Nano.

[26] Streit J. K., Park K., Ku Z., Yi Y.-J., Vaia R. A. (2021). Tuning
Hierarchical Order and Plasmonic Coupling of Large-Area, Polymer-Grafted
Gold Nanorod Assemblies via Flow-Coating. ACS
Appl. Mater. Interfaces.

[27] Lo
Verso F., Yelash L., Egorov S. A., Binder K. (2012). Effect of
the Solvent Quality on the Structural Rearrangement of Spherical Brushes:
Coarse-Grained Models. Soft Matter.

[28] Bachhar N., Jiao Y., Asai M., Akcora P., Bandyopadhyaya R., Kumar S. K. (2017). Impact of the Distributions
of Core Size and Grafting
Density on the Self-Assembly of Polymer Grafted Nanoparticles. Macromolecules.

[29] Akcora P., Liu H., Kumar S. K., Moll J., Li Y., Benicewicz B. C., Schadler L. S., Acehan D., Panagiotopoulos A. Z., Pryamitsyn V., Ganesan V., Ilavsky J., Thiyagarajan P., Colby R. H., Douglas J. F. (2009). Anisotropic Self-Assembly
of Spherical
Polymer-Grafted Nanoparticles. Nat. Mater..

[30] Martin T. B., Mongcopa K. I. S., Ashkar R., Butler P., Krishnamoorti R., Jayaraman A. (2015). Wetting–Dewetting
and Dispersion–Aggregation
Transitions Are Distinct for Polymer Grafted Nanoparticles in Chemically
Dissimilar Polymer Matrix. J. Am. Chem. Soc..

[31] Koerner H., Drummy L. F., Benicewicz B., Li Y., Vaia R. A. (2013). Nonisotropic
Self-Organization of Single-Component Hairy Nanoparticle Assemblies. ACS Macro Lett..

[32] Ethier J. G., Hall L. M. (2018). Structure and Entanglement Network of Model Polymer-Grafted
Nanoparticle Monolayers. Macromolecules.

[33] Choi J., Hui C. M., Schmitt M., Pietrasik J., Margel S., Matyjazsewski K., Bockstaller M. R. (2013). Effect
of Polymer-Graft Modification on the Order Formation in Particle Assembly
Structures. Langmuir.

[34] Ginzburg V. V. (2017). Modeling
the Morphology and Phase Behavior of One-Component Polymer-Grafted
Nanoparticle Systems. Macromolecules.

[35] Bockstaller M. R., Lapetnikov Y., Margel S., Thomas E. L. (2003). Size-Selective Organization
of Enthalpic Compatibilized Nanocrystals in Ternary Block Copolymer/Particle
Mixtures. J. Am. Chem. Soc..

[36] Pérez-Lemus G.
R., Armas-Pérez J. C., Mendoza A., Quintana-H J., Ramírez-Hernández A. (2019). Hierarchical
Complex Self-Assembly
in Binary Nanoparticle Mixtures. J. Phys.: Condens.
Matter.

[37] Bae Y. C., Lambert S. M., Soane D. S., Prausnitz J. M. (1991). Cloud-Point
Curves of Polymer Solutions from Thermooptical Measurements. Macromolecules.

[38] Park J.-S., Kataoka K. (2006). Precise Control of Lower Critical Solution Temperature
of Thermosensitive Poly­(2-Isopropyl-2-Oxazoline) via Gradient Copolymerization
with 2-Ethyl-2-oxazoline as a Hydrophilic Comonomer. Macromolecules.

[39] Garas G., Kosmas M. (1994). Effect of Chain Architecture on the Cloud Point Curves
of Binary Blends of Star Polymers. Macromolecules.

[40] Turkevich J., Stevenson P. C., Hillier J. (1951). A Study of the Nucleation and Growth
Processes in the Synthesis of Colloidal Gold. Discuss. Faraday Soc..

[41] Oliveira A. E. F., Pereira A. C., Resende M. A. C., Ferreira L. F. (2023). Gold Nanoparticles:
A Didactic Step-by-Step of the Synthesis Using the Turkevich Method,
Mechanisms, and Characterizations. Analytica.

[42] Dong J., Carpinone P. L., Pyrgiotakis G., Demokritou P., Moudgil B. M. (2020). Synthesis of Precision
Gold Nanoparticles Using Turkevich
Method. KONA Powder and Particle Journal.

[43] Liu X., Atwater M., Wang J., Huo Q. (2007). Extinction Coefficient
of Gold Nanoparticles with Different Sizes and Different Capping Ligands. Colloids Surf., B.

[44] Benoit D. N., Zhu H., Lilierose M. H., Verm R. A., Ali N., Morrison A. N., Fortner J. D., Avendano C., Colvin V. L. (2012). Measuring the Grafting
Density of Nanoparticles in Solution by Analytical Ultracentrifugation
and Total Organic Carbon Analysis. Anal. Chem..

[45] Ranoszek-Soliwoda K., Girleanu M., Tkacz-Szczesna B., Rosowski M., Celichowski G., Brinkmann M., Ersen O., Grobelny J. (2016). Versatile Phase Transfer
Method for the Efficient Surface Functionalization of Gold Nanoparticles:
Towards Controlled Nanoparticle Dispersion in a Polymer Matrix. J. Nanomater..

[46] He Y. Q., Liu S. P., Kong L., Liu Z. F. (2005). A Study on the Sizes
and Concentrations of Gold Nanoparticles by Spectra of Absorption,
Resonance Rayleigh Scattering and Resonance Non-Linear Scattering. Spectrochimica Acta Part A: Molecular and Biomolecular Spectroscopy.

[47] Yockell-Lelièvre H., Gingras D., Vallée R., Ritcey A. M. (2009). Coupling of Localized
Surface Plasmon Resonance in Self-Organized Polystyrene-Capped Gold
Nanoparticle Films. J. Phys. Chem. C.

[48] Underwood S., Mulvaney P. (1994). Effect of the Solution
Refractive Index on the Color
of Gold Colloids. Langmuir.

[49] Lee J., Bae C., Ou Z., Park S., Kim J., Kim J. (2021). Nanoscopic
Morphological Effect on the Optical Properties of Polymer-Grafted
Gold Polyhedra. Nanoscale Advances.

[50] Alexander S. (1977). Adsorption
of Chain Molecules with a Polar Head a Scaling Description. J. Phys. (Paris).

[51] de
Gennes P. G. (1980). Conformations of Polymers Attached to an Interface. Macromolecules.

[52] Hore M. J. A., Ford J., Ohno K., Composto R. J., Hammouda B. (2013). Direct Measurements
of Polymer Brush Conformation Using Small-Angle Neutron Scattering
(SANS) from Highly Grafted Iron Oxide Nanoparticles in Homopolymer
Melts. Macromolecules.

[53] Dukes D., Li Y., Lewis S., Benicewicz B., Schadler L., Kumar S. K. (2010). Conformational
Transitions of Spherical Polymer Brushes: Synthesis, Characterization,
and Theory. Macromolecules.

[54] Ohno K., Morinaga T., Takeno S., Tsujii Y., Fukuda T. (2007). Suspensions
of Silica Particles Grafted with Concentrated Polymer Brush: Effects
of Graft Chain Length on Brush Layer Thickness and Colloidal Crystallization. Macromolecules.

[55] Wijmans C. M., Zhulina E. B. (1993). Polymer Brushes at Curved Surfaces. Macromolecules.

[56] Saeki S., Kuwahara N., Konno S., Kaneko’ M. (1973). Upper and
Lower Critical Solution Temperatures in Polystyrene Solutions. Macromolecules.

[57] Higgins J. S., Fruitwala H., Tomlins P. E. (1989). Correlation of Phase-Separation Behavior
in Polymer Blends with Thermodynamic Measurements in the One-Phase
Region. Macromolecules.

[58] Schwahn D., Janssen S., Springer T. (1992). Early State
of Spinodal Decomposition
Studied with Small Angle Neutron Scattering in the Blend Deuteropolystyrene
and Polyvinylmethylether: A Comparison with the Cahn–Hilliard–Cook
Theory. J. Chem. Phys..

[59] Witten T. A., Sander L. M. (1983). Diffusion-Limited Aggregation.
Physical Review B.

[60] Weitz D. A., Huang J. S., Lin M. Y., Sung J. (1985). Limits of
the Fractal
Dimension for Irreversible Kinetic Aggregation of Gold Colloids. Phys. Rev. Lett..

[61] Weitz D. A., Lin M. Y., Sandroff C. (1985). Colloidal Aggregation
Revisited:
New Insights Based on Fractal Structure and Surface-Enhanced Raman
Scattering. Surf. Sci..

[62] Lin M. Y., Lindsay H. M., Weitz D. A., Ball R. C., Klein R., Meakin P. (1989). Universality in Colloid
Aggregation. Nature.

[63] Jaeger G. (1998). The Ehrenfest
Classification of Phase Transitions: Introduction and Evolution. Archive for History of Exact Sciences.

[64] Khasat N., Pennisi R. W., Hadjichristidis N., Fetters L. J. (1988). Dilute Solution
Behavior of 3-Arm Asymmetric and Regular 3- and 12-Arm Polystyrene
Stars. Macromolecules.

[65] Roovers J., Toporowski P. M. (1983). Synthesis
of High Molecular Weight Ring Polystyrenes. Macromolecules.

[66] Roovers J. (1985). Dilute-Solution
Properties of Ring Polystyrenes. J. Polym. Sci.,
Polym. Phys. Ed..

[67] Suzuki J., Takano A., Matsushita Y. (2011). The Theta-Temperature
Depression
Caused by Topological Effect in Ring Polymers Studied by Monte Carlo
Simulation. J. Chem. Phys..

[68] Narros A., Moreno A. J., Likos C. N. (2013). Effects of Knots on Ring Polymers
in Solvents of Varying Quality. Macromolecules.

[69] Orofino T. A., Wenger F. (1963). Dilute Solution Properties
of Branched Polymers. Polystyrene
Trifunctional Star Molecules. J. Phys. Chem..

[70] Mirzaev S. Z., Heimburg T., Kaatze U. (2010). Critical Behavior
of Polystyrene-Cyclohexane:
Heat Capacity and Mass Density. Phys. Rev. E.

[71] Meakin P. (1983). Formation
of Fractal Clusters and Networks by Irreversible Diffusion-Limited
Aggregation. Phys. Rev. Lett..

[72] Kolb M., Botet R., Jullien R. (1983). Scaling of
Kinetically Growing Clusters. Phys. Rev. Lett..

[73] Swislow G., Sun S. T., Nishio I., Tanaka T. (1980). Coil-Globule Phase
Transition in a Single Polystyrene Chain in Cyclohexane. Phys. Rev. Lett..

[74] Stepanek P., Konak C., Sedlacek B. (1982). Coil-Globule Transition
of a Single
Polystyrene Chain in Dioctyl Phthalate. Macromolecules.

[75] Zhulina E. B., Borisov O. V., Pryamitsyn V. A., Birshtein T. M. (1991). Coil-Globule
Type Transitions in Polymers. 1. Collapse of Layers of Grafted Polymer
Chains. Macromolecules.

[76] Pomposo J. A., Perez-Baena I., Lo Verso F., Moreno A. J., Arbe A., Colmenero J. (2014). How Far Are
Single-Chain Polymer Nanoparticles in Solution
from the Globular State?. ACS Macro Lett..

